# The origin of incipient ferroelectricity in lead telluride

**DOI:** 10.1038/ncomms12291

**Published:** 2016-07-22

**Authors:** M. P. Jiang, M. Trigo, I. Savić, S. Fahy, É. D. Murray, C. Bray, J. Clark, T. Henighan, M. Kozina, M. Chollet, J. M. Glownia, M. C. Hoffmann, D. Zhu, O. Delaire, A. F. May, B. C. Sales, A. M. Lindenberg, P. Zalden, T. Sato, R. Merlin, D. A. Reis

**Affiliations:** 1 Stanford PULSE Institute, SLAC National Accelerator Laboratory, Menlo Park, California 94025, USA; 2 Stanford Institute for Materials and Energy Sciences, SLAC National Accelerator Laboratory, Menlo Park, California 94025, USA; 3 Department of Physics, Stanford University, Stanford, California 94305, USA; 4 Tyndall National Institute, Lee Maltings Complex, Dyke Parade, Cork T12R5CP, Ireland; 5 Department of Physics, University College Cork, College Road, Cork, Ireland; 6 Departments of Physics and Materials, Imperial College London, London SW7 2AZ, UK; 7 Department of Applied Physics, Stanford University, Stanford, California 94305, USA; 8 Linac Coherent Light Source, SLAC National Accelerator Laboratory, Menlo Park, California 94025, USA; 9 Department of Mechanical Engineering and Materials Science, Duke University, Durham, North Carolina 27708, USA; 10 Materials Science and Technology Division, Oak Ridge National Laboratory, Oak Ridge, Tennessee 37831, USA; 11 Department of Materials Science and Engineering, Stanford University, Stanford, California 94305, USA; 12 RIKEN SPring-8 Center, Kouto 1-1-1, Sayo, Hyogo 679-5148, Japan; 13 Department of Chemistry, The School of Science, The University of Tokyo, 7-3-1 Hongo, Bunkyo-ku, Tokyo 113-0033, Japan; 14 Department of Physics, University of Michigan, Ann Arbor, Michigan 48109, USA

## Abstract

The interactions between electrons and lattice vibrations are fundamental to materials behaviour. In the case of group IV–VI, V and related materials, these interactions are strong, and the materials exist near electronic and structural phase transitions. The prototypical example is PbTe whose incipient ferroelectric behaviour has been recently associated with large phonon anharmonicity and thermoelectricity. Here we show that it is primarily electron-phonon coupling involving electron states near the band edges that leads to the ferroelectric instability in PbTe. Using a combination of nonequilibrium lattice dynamics measurements and first principles calculations, we find that photoexcitation reduces the Peierls-like electronic instability and reinforces the paraelectric state. This weakens the long-range forces along the cubic direction tied to resonant bonding and low lattice thermal conductivity. Our results demonstrate how free-electron-laser-based ultrafast X-ray scattering can be utilized to shed light on the microscopic mechanisms that determine materials properties.

## Abstract (web summary)

Group IV–VI materials often exist in a state near an electronic or structural phase transition. Here, the authors use ultrafast X-ray scattering to show that coupling of band-edge electrons and phonons causes the ferroelectric instability observed in lead telluride.

## Graphical abstract

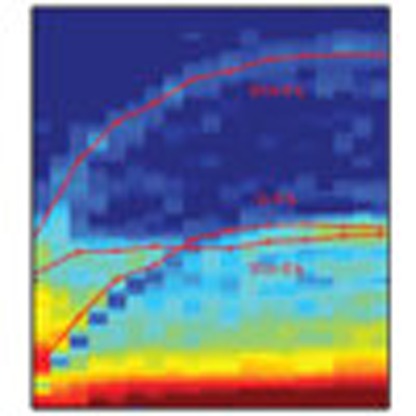

The narrow gap IV–VI semiconductors and group V semimetals are characterized by coupled electronic and lattice instabilities^1^,^2^,^3^,^4^,^5^. The general behaviour has been understood at least qualitatively in terms of electron-phonon interactions associated with the unsaturated p-like bonding in these materials. In the case of PbTe, a ferroelectric instability develops with decreasing temperature, but the material remains in the paraelectric, rock-salt phase to the lowest temperatures measured^6^,^7^.

Recent measurements^8^,^9^,^10^,^11^ on PbTe reveal anomalous lattice dynamics with increasing temperature, especially in the soft transverse optical (TO) phonon branch associated with the ferroelectric instability. This has been interpreted in terms of both giant anharmonicity^8^,^12^,^13^,^14^,^15^,^16^ and local symmetry breaking due to off-centring of the Pb ions^9^,^10^. The observed anomalies have prompted renewed theoretical and computational interest. Much of this work has concentrated on the role of lattice anharmonicity and phonon-phonon interactions^12^,^13^,^14^,^15^,^16^,^17^,^18^,^19^. On the other hand, a few theoretical studies have focused on the importance of electron-phonon coupling, for example, in the link between the mixed ionic-covalent character of Pb–Te bonds and the soft mode behaviour^5^,^16^ or the large polarizability of the Pb ion's lone 6s^2^ pair^20^,^21^. The instability has also been discussed in terms of the large polarizability of resonant valence p-like bonds^3^,^4^, which has recently been associated with long-range interactions, the low lattice thermal conductivity, and the high thermoelectric performance^22^. Thus, the origin of the soft phonon branch and the associated anharmonicity in PbTe remains controversial, partially due to the difficulty of separating the coupled electronic and lattice interactions experimentally.

The incipient ferroelectric behaviour of IV–VI compounds is strongly sensitive to carrier concentration^23^, which suggests the importance of the valence and conduction p-type bands very near the band edges in determining the equilibrium structure^1^. Ultrafast photoexcitation provides a means to affect the electron-phonon coupling by redistributing the carrier population near the band edges at constant volume and without introducing defects associated with doping. Our motivation for the present work is to use a combination of ultrafast X-ray scattering and first principles calculations to isolate the role of electron-phonon interactions in photoexcited PbTe.

In particular, differential changes in the momentum-dependent phonon-phonon displacement correlations can be accessed via Fourier-transform inelastic X-ray scattering (FT-IXS)^24^,^25^. In this technique, an ultrafast pump pulse is used to suddenly photoexcite carriers on a time scale that is fast compared with all the relevant phonon periods. In turn, the modification of the band-population renormalizes the phonon frequencies and, in principle, the eigenvectors as well, mixing the acoustic and optical branches. The projection of the initially thermally occupied vibrational modes onto these new modes produces a nonequilibrium state where the displacement correlations evolve in time. This method thus allows us to extract explicit information about the coupling of the electronic states near the energy gap with lattice vibrations. In previous experiments on photoexcited Ge^24^,^25^, temporal oscillations in the X-ray diffuse scattering were observed, corresponding to correlations between phonons of equal and opposite momenta belonging to the same branch and oscillating at twice the phonon frequency.

Here we measure oscillations in momentum-dependent phonon–phonon displacement correlations for PbTe excited across its direct band gap. In addition to oscillations at twice the transverse acoustic phonon frequency, we measure the sum and difference frequencies for phonons of different branches. These combination modes result from a sudden inhomogeneous change in the interatomic forces and the dynamical matrices on photoexcitation. Our simulations, based on *ab initio* calculations, reproduce the experimentally observed combination modes due to a weakening of the long-range interactions along the bonding directions. This inhomogeneous change in forces is a direct consequence of the redistribution of the population of electronic states near the direct band-gap at the L-point that also results in a stabilization of the paraelectric phase. Thus, we demonstrate that the particular coupling of electronic states near the band edges via the zone centre TO mode is the primary mechanism that induces the TO mode softening that drives the incipient ferroelectric behaviour. Our results are consistent with the resonant bonding picture of the soft mode behaviour, verifying that it is primarily the electronic states near the energy gap that are responsible for this effect. These findings open new opportunities for the development of more efficient thermoelectric materials (for example, by controlling the identified mechanism for incipient ferroelectricity via alloying) and contribute to the general understanding of mechanisms for ferroelectric phase transitions.

## Results

### Identification of combination modes in FT-IXS spectra

The experiments were performed at room temperature using 50 fs, 8.7 keV X-rays from the X-ray pump probe instrument at the Linac Coherent Light Source (LCLS)^26^ free-electron laser. We excite ∼2 × 10^20^ cm^−3^ carriers per L-valley (∼0.5% valence excitation) across the direct band gap of low-carrier-density (4 × 10^17^ cm^−3^) n-type PbTe with 60 fs infrared pump pulses with central wavelength of 2 μm (see Methods section). Subsequently, we track the X-ray diffuse scattering as a function of time delay τ between the infrared pump and X-ray probe pulses, referencing differential changes in the scattering intensity with and without the photoexcitation. Absorption of the pump pulse produces correlated pairs of phonons with equal and opposite momenta that are probed by femtosecond X-ray diffuse scattering. The intensity of this scattering is proportional to snapshots of the equal-time phonon-phonon displacement correlations as a function of momentum transfer^24^. The amplitudes of the temporal oscillatory components are directly related to the strength of the interaction of the phonons with the photoexcited electron density, through changes in the dynamical matrices^27^.

**Figure 1:**
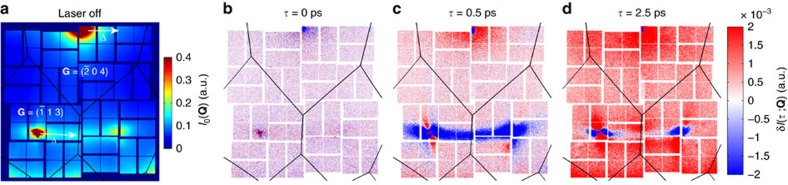
Femtosecond X-ray diffuse scattering from lead telluride. (**a**) Reference scattering from PbTe before photoexcitation. The overlaid black lines represent the Brillouin zone boundaries and two zones of interest, **G**=(1 3) and **G**=(0 4), are labelled. The white arrows point along the respective high-symmetry **Δ** (**Γ** to **X**) wavevector directions in each zone. The intensity of the X-ray scattering in this frame is denoted as *I*_0_(**Q**) and in arbitrary units (a.u.). (**b**–**d**) Differential scattering *δI*(*τ*;**Q**)=*I*(*τ*;**Q**)−*I*_0_(**Q**), in which the reference frame is subtracted from the pattern at each time delay *τ* following ultrafast infrared excitation. The time delays shown here are *τ*=0, 0.5 and 2.5 ps. Within 0.5 ps, a strong decrease in intensity is seen along the **Δ** line in the **G**=(1 3) BZ while an increase in intensity is seen along **Δ** in the **G**=(0 4) BZ.

Figure 1 presents two-dimensional X-ray scattering images from PbTe for a fixed crystal configuration. Each pixel corresponds to a different momentum transfer **Q**. Figure 1a shows the average diffuse scattering intensity *I*_0_(**Q**), from the unexcited state, that is, when the pump arrives after the X-ray probe (*τ*<0). The overlaid black lines represent the boundary between different Brillouin zones (BZ) for the fcc Bravais lattice. Two BZs of interest, **G**=(1 3) and **G**=(0 4), are labelled in reciprocal lattice units (rlu) of 2*π*/*a*_0_, where *a*_0_ is the length of a side of the conventional cubic cell. The white arrows point along the reduced wave vectors **q**=**Q**−**G**, where in this case **q**≈(0 *q*_y_ 0), that is, it is approximately along the high-symmetry **Δ** (**Γ** to **X**) direction in each zone. Figure 1b–d are differential scattering intensity patterns *δI*(*τ*;**Q**)=*I*(*τ*;**Q**)−*I*_0_(**Q**), in which the unexcited state frame (Fig. 1a) is subtracted from the pattern at each time delay. The time delays shown here are *τ*=0, 0.5 and 2.5 ps. Increases in the scattering intensity relative to the reference frame are marked in red while decreases in the intensity are shown in blue. While at the moment of excitation (*τ*=0 ps) there are not yet significant changes in scattering intensity, within 0.5 ps, a strong decrease in intensity is seen along the **Δ** direction in the **G**=(1 3) BZ while an increase in intensity is seen along **Δ** in the **G**=(0 4) BZ. By 2.5 ps, the diffuse scattering shows a general increase and the prominent decrease along the **Δ** line in the (1 3) BZ has largely subsided.

**Figure 2:**
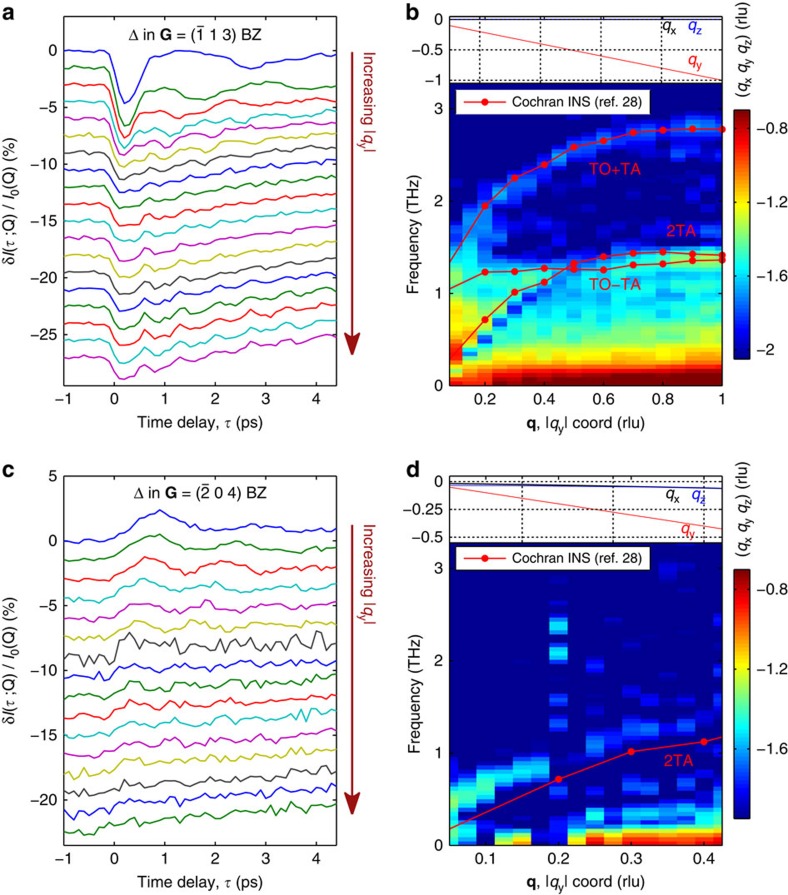
Fourier-transform inelastic X-ray scattering (FT-IXS) spectra for transverse two-phonon excitations along the bonding direction. (**a**,**c**) Time-domain differential X-ray diffuse scattering intensities at various *q* coordinates along the approximate **Γ** to **X** (**Δ**) high-symmetry reduced wavevector direction in both the (1 3) Brillouin zone (BZ) and the (0 4) BZ respectively. This direction is shown in both BZs in Fig. 1a. The observed modulations stem from temporal coherences in the momentum-dependent phonon-phonon correlations. (**b**,**d**) Simultaneous frequency and momentum representations of the time-domain data in **a**,**c**, respectively, on a base-10 logarithmic scale, with two-phonon dispersion calculated from the INS data of ref. 28 overlaid. The trajectories of the momentum coordinates are displayed above each spectrum in reciprocal lattice units (rlu). Note the deviation of the *q*_x_ and *q*_z_ coordinates from the nominal Δ wavevector coordinates in panel (**d**). Clear matches with first overtone and combination transverse polarized phonon states are seen. Note that broadband behaviour seen near |*q*_y_|∼0.2 in panel **d** is due to noisier statistics that arise from an average over a smaller solid angle, in comparison with data at other *q* coordinates.

Figure 2 shows the relative differential changes *δI*(*τ*;**Q**)/*I*_*0*_(**Q**) along the two **Δ** lines indicated in Fig. 1, in the time domain (Fig. 2a,c) and the frequency domain (Fig. 2b,d). The precise trajectory for the reduced wave vectors (*q*_*x*_ *q*_*y*_ *q*_*z*_) is shown in the upper portion of Fig. 2b,d. In our experimental geometry, the diffuse scattering along **Δ** is sensitive primarily to transverse phonons polarized along (0 0 1). From top to bottom the data in Fig. 2a span from near zone-centre to zone edge in the **G**=(1 3) BZ and in Fig. 2c from near zone centre to approximately half the distance to zone edge in the **G**=(0 4) BZ. The data in the () zone show a rapid decrease in scattering followed by damped oscillatory behaviour consisting of multiple frequencies that disperse with increasing |*q*_y_| on top of a several picosecond-scale increase. Meanwhile in the (0 4) zone, the data show an increase in scattering with damped oscillations.

In Fig. 2b,d, we plot the Fourier transform of the time-domain data for the (1 3) and (0 4) zones as a function of |*q*_y_|. Several dispersive features are immediately apparent in the spectra on top of a broad and relatively featureless low-frequency background originating from the slow variations in the diffuse scattering over time. The dispersive features in Fig. 2b match well with the frequencies expected for the first transverse acoustic overtone (2TA) and TO and acoustic combination modes (TO±TA) as extracted from the INS data reported by Cochran *et al*.^28^ (overlaid in red). The overtone is due to phonon-phonon correlations between modes at +**q** and −**q** within the same branch^24^, while the combination modes originate from correlations between different branches. These mixed terms were not observed in previous FT-IXS measurements^24^,^25^. Photoexcitation can lead to combination modes only through inhomogeneous changes in the various elements of the dynamical matrix, mixing the eigenvectors. The predominance of the combination modes indicates that photoexcitation couples the TO and TA eigenvectors in the nonequilibrium state and does not just renormalize their frequencies as would occur through an overall scaling of the dynamical matrix. This mixing of the branches is consistent with a picture where photoexcitation primarily affects the long-range interactions that have been linked to the ferroelectric instability through resonant bonding^22^. As shown below, our first principles calculations support this picture while also pinpointing the associated electronic states that are responsible for instability.

In the (0 4) BZ, the sensitivity to optical phonons is low and we therefore only expect to see overtones of the acoustic modes, consistent with the results shown in Fig. 2d. Here we attribute the dispersion to the 2TA overtone. The discrepancy with the neutron results (red line) is likely due to systematic deviations from the **Δ**-line for this zone in our geometry (finite *q*_x_ and *q*_z_; see coordinate trajectory in the figure and Methods section).

### First principles simulation of photoexcited PbTe

To simulate the experimental conditions, we calculate the time evolution of the displacement correlations between phonon modes due to a sudden promotion of valence electrons to the conduction band^27^. The initial values of these displacement correlations for unexcited PbTe were calculated using density functional theory (DFT, see Methods section). We calculate the interatomic force constants of photoexcited PbTe using constrained density functional theory (CDFT)^29^, taking that 0.5% of valence electrons are promoted into the conduction band, as estimated in the experiments (see Methods section). In the CDFT calculation, we assume that electron and hole populations thermalize rapidly within their respective excited-state bands according to Fermi-Dirac distributions, which is reasonable since the typical electron-electron scattering times are on the order of tens of femtoseconds. This scattering time and the excitation pulse itself are much shorter than the shortest phonon period. Thus, for the simulations we consider the photoexcitation as sudden.

**Figure 3:**
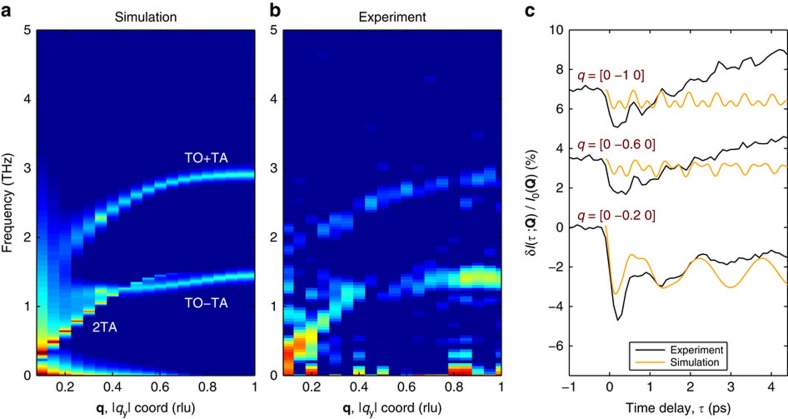
Simulation and experimental results show combination and overtone modes. (**a**) Simulated FT-IXS spectrum along the **Δ** line in the (1 3) BZ. The 2TA and TO±TA modes are labelled. The spectrum qualitatively matches the experimental data in **b**. (**b**) Experimental FT-IXS spectrum along the same high-symmetry direction in the (1 3) BZ seen in Fig. 2b and with the slowly varying low frequency background filtered. (**c**) Comparison of experimental time-domain signal (black traces, unfiltered) at select wavevector coordinates along **Δ** as taken from Fig. 2a with calculated signal (orange traces). There is a good qualitative match between experimental and model results, notably in the strong initial dip seen at *τ*=0, connected to a considerable hardening of the TO branch frequencies.

A sudden change of interatomic force constants before and after photoexcitation leads to the time evolution of the displacement correlations between phonon modes, which was derived starting from the equations of motion of damped harmonic oscillators for each phonon mode^27^. The time-dependent X-ray diffuse scattering signal is proportional to those displacement correlations^24^,^27^ and its Fourier transform gives the simulated FT-IXS spectrum along **Δ** in the (1 3) BZ shown in Fig. 3a. The FT-IXS spectrum calculation makes use of the excited-state harmonic and anharmonic (3^rd^ order) force constants computed using CDFT, as well as the ground-state harmonic force constants computed using DFT that characterize the initial thermal state. Here the anharmonic force constants are only used to introduce damping. To better compare the simulation and experiments, we filter the experimental data to remove the slowly varying background before taking the Fourier transform, as shown in Fig. 3b. Both combination modes (TO±TA) and an overtone mode (2TA) appear in the calculations and are labelled. The simulation matches the experimental data, capturing many of its general features and thus reflecting the closeness of the model in depicting the effects of ultrafast photoexcitation on the material.

**Figure 4:**
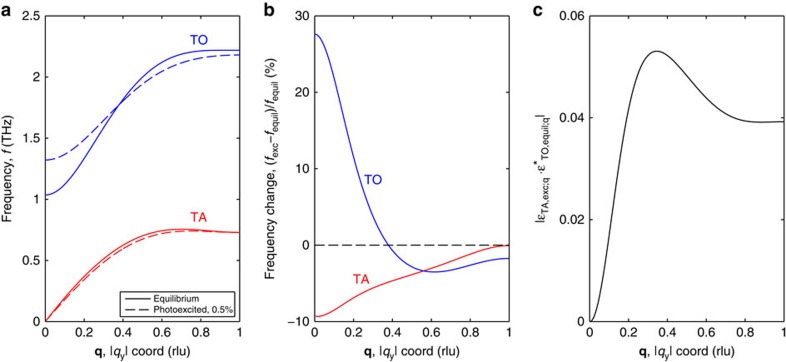
Comparison of calculated transverse optical (TO) and transverse acoustic (TA) dispersions at equilibrium and under 0.5% photoexcitation. (**a**) Calculated dispersions (TA in red and TO in blue) for PbTe along the **Δ** direction (marked in reciprocal lattice units, rlu) in the (1 3) zone at both equilibrium (DFT, solid lines) and with 0.5% photoexcitation (CDFT, dashed lines). (**b**) Relative (percentile) changes in frequency between the DFT and CDFT calculations for both the TO (blue) and TA (red) phonon branches along the **Δ**. The TA modes experience a softening in frequency across the entire wavevector direction on photoexcitation whereas the TO modes harden dramatically near zone centre and softens near zone edge. (**c**) Projection of the calculated excited state TA branch eigenvector onto the calculated equilibrium state TO branch eigenvector along the same **Δ** line. The projection represents the degree of equilibrium TO character that has been translated into the TA modes on photoexcitation and thus the amount of mode-mixing that has occurred. Note that such renormalization of the frequencies and eigenvectors will only result in excitation of two-phonon overtone and combination modes if it is sudden compared with the phonon period.

**Figure 5:**
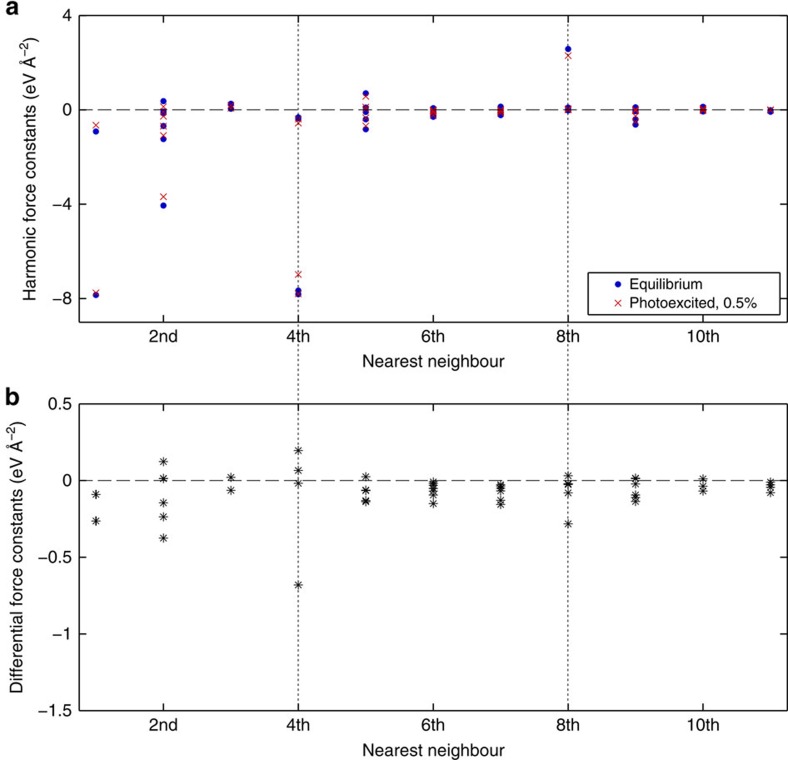
Comparison of calculated harmonic interatomic force constants at equilibrium and under 0.5% photoexcitation. (**a**) Nonzero harmonic interatomic force constants (IFCs) calculated for both equilibrium (blue dots) and 0.5% photoexcited PbTe (red crosses) on 216-atom supercells. The IFCs for the first eleven nearest neighbouring atoms are displayed. Particularly large IFCs are observed between first, fourth and eighth nearest neighbours, which correspond to interactions along the <100> bonding direction responsible for PbTe's incipient ferroelectricity. See Fig. 6 for a real-space depiction of these neighbours. The absolute values of these large IFCs notably decrease with photoexcitation, driving PbTe further away from the ferroelectric phase. (**b**) Differences between the absolute values of the equilibrium and photoexcited IFCs in **a**.

**Figure 6:**
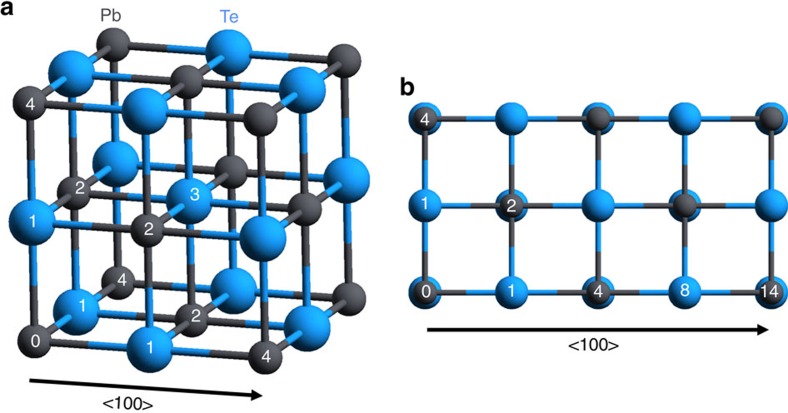
Nearest neighbors within cubic rocksalt structure. (**a**) Three-dimensional depiction of crystal structure. (**b**) Overhead depiction of crystal structure with an emphasis on the <100> cubic direction.

Figure 3c shows the experimental (black lines, unfiltered) and calculated (orange lines) time-dependent diffuse scattering at select coordinates along **Δ** in the (1 3) BZ. Note that the model calculations do not account for relaxation of the photoexcited carriers or the heating of the lattice, which could explain the picosecond rise seen in the data but not the model. Importantly, the strong initial dip and phase of the oscillation observed in the experiment is well reproduced by the model. In the calculations, this coincides with substantial hardening of the TO phonon branch of up to ∼28% at **Γ** (Fig. 4a,b), accompanied with a distinctive weakening of long-range interactions involving both 4^th^ and 8^th^ nearest neighbours (Fig. 5). These particular force constants correspond to interactions with the second and third neighbours along the <100> bonding direction in the cubic crystal structure of PbTe (see Fig. 6). The calculations reproduce the anomalously strong long-range interactions that are expected along this direction that have been attributed to resonant bonding involving *p*-band valence electrons^22^. It is further observed that the experimental lifetimes seen in Fig. 3c appear shorter than those predicted in the simulations.

It is especially important to note here that the appearance of combination modes is a direct indication of the nonequilibrium state following photoexcitation and could only appear on a sudden inhomogeneous change in the interatomic forces. To illustrate the degree of mixing we show in Fig. 4c the projection of the calculated TA eigenvectors of the excited state onto those of the equilibrium TO state, 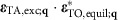, along the **Δ** line. Here the projection represents the degree of equilibrium TO character that has been mixed into the TA modes on photoexcitation. Such mode mixing directly leads to the experimentally observed combination modes seen in Figs 2 and 3.

The general agreement between the measured and simulated two-phonon FT-IXS spectra indicates that photoexcitation of PbTe stabilizes the paraelectric phase, as described below. The specific observation of combination modes along the **Δ** line is consistent with differential changes in the real-space force constants, as obtained in the calculations. Furthermore, the initial rise of the oscillation after *τ*=0 in the (0 4) BZ (Fig. 2c) is consistent with a sudden softening of the TA branch frequency on photoexcitation. This conclusion can be drawn independently of the calculations since the square of the phonon displacements, and thus the diffuse scattering, is inversely proportional to the phonon frequency^24^. In contrast, the initial dip of the response in the (1 3) BZ (Fig. 2a) likely originates from a hardening of the TO branch near zone centre after photoexcitation, as predicted by calculations (see Fig. 4b). Furthermore, we note that the absence of a clear signal at 2TO in the (1 3) BZ in the experiment is consistent with the simulation results, in which the square of the phonon displacement dampens at twice the rate of the already strongly anharmonic TO mode.

## Discussion

**Figure 7:**
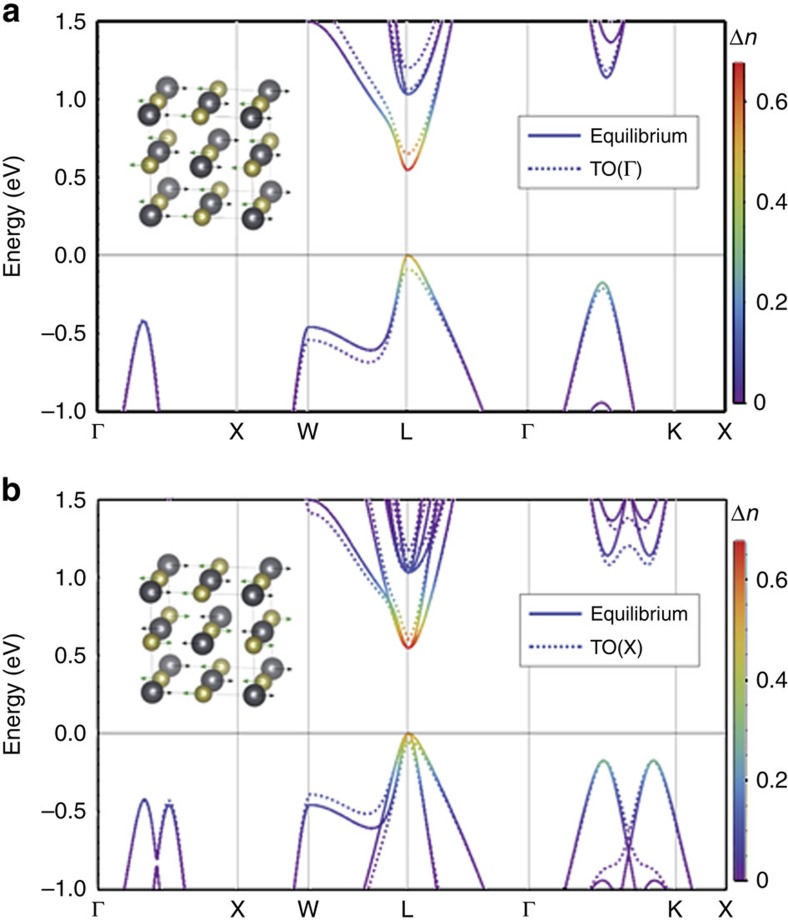
Effect of transverse optical (TO) phonon displacements on the electronic band structure. (**a**) A TO phonon displacement at **Γ** induces a notable separation of the lowest conduction and highest valence bands (dashed) in comparison with the equilibrium configuration (solid). The colour shading of the bands indicates the change of occupation of electron and hole states following photoexcitation. The band shifts occur most dramatically within a region of reciprocal space limited along the **L**–**W** line. The widening of the gap is consistent with a Jones-Peierls distortion mechanism, demonstrating an inclination towards the lower-symmetry rhombohedral ferroelectric structure. Photoexcitation reduces the effectiveness of this mechanism, resulting in a hardening of the TO frequency near zone centre. (**b**) Separately, a TO phonon displacement at **X** results in a notable closing of the gap between the bottommost conduction and topmost valence bands (dashed) relative to the ground state configuration (solid). Once again, the activity is along the **L**–**W** line. In contrast with zone centre, photoexcitation results in a softening of the TO frequency here.

The origin of the photo-induced stabilization of the paraelectric phase of PbTe can be understood from the following arguments. Figure 7 shows the highest valence and lowest conduction bands of photoexcited PbTe, calculated with and without displacement of the atoms corresponding to the soft TO phonon at zone centre (**Γ**-point) (Fig. 7a) and at the **X**-point (Fig. 7b). The solid line shows the bands for the equilibrium atomic positions and the dashed lines show the bands with the TO phonon mode frozen in. The colour shading of the bands indicates the change of occupation of electron and hole states following photoexcitation (see Methods section). In Fig. 7a, we see that the zone centre TO(**Γ**) phonon displacement lowers the energy of states near the valence band maximum and increases the energy of states near the conduction band minimum, particularly along the **L**–**W** line. This is in accordance with the Jones-Peierls distortion mechanism, which stabilizes the rhombohedral structure of the group V elements and the ferroelectrically-distorted IV–VI compounds by coupling the low-lying conduction bands with the higher lying valence bands in the (111) face of the fcc Brillouin zone^2^. The underlying effect of electron-phonon coupling here is the same as that of the Kohn anomaly in three-dimensional metals and the Peierls instability of one-dimensional metals. This explains the strong photo-induced softening of the zone centre A_1g_ mode in the group V semimetal bismuth^30^,^31^, where photoexcitation reduces the Peierls distortion and drives the system closer to the symmetric phase. Similarly, in the case of PbTe, a relatively small change of the occupation of these bands (filling the lower conduction bands and vacating the highest valence bands) reduces the effectiveness of the TO displacement in lowering the total electronic energy. The result is an increase in the soft-TO mode frequency at **Γ**, as observed in calculations for PbTe (Fig. 4b). In contrast, the TO mode at **X** (Fig. 7b) strongly couples the valence bands causing them to repel along **L**–**W**, shifting the highest energy band upwards under phonon displacements. Consequently, in PbTe photoexcitation gives rise to a softening of the TO frequency at **X** (Fig. 4b) at the same time that it reduces the soft-mode behaviour with the near **Γ** hardening.

The interatomic forces, particularly the aforementioned long-range interatomic terms with 4th and 8th neighbours, can be understood in terms of these strong phonon wavevector-dependent frequency shifts and their coupling with the conduction and valence bands near the energy gap. Via Fourier transform, this momentum dependent phonon frequency renormalization directly translates to the prominent long-range real-space coupling noted in ref. 22. By promoting the carriers from valence to conduction band, the wavevector-dependent renormalization of phonon frequencies due to the electron-phonon coupling becomes weaker^32^, resulting in shorter-range interatomic forces (see Fig. 5). Our measurements and calculations of photoexcited PbTe demonstrate that the electronic states very near the direct gap dominate this mechanism and that the effect on mode frequencies depends strongly on the phonon wavevector.

We note that the strong electron-TO(**Γ**) phonon coupling occurs between bands that originate from half-filled degenerate states in the absence of ionicity^2^. This is compatible with the resonant bonding picture^22^,^33^, which argues that the zone centre optical-phonon displacement tends to lower the energies of the half-saturated *p*-bonds on one side of the atom relative to the other^5^, leading to its strong coupling with the electronic bands. However, the band picture provides more detailed insights into the phonon-momentum-dependence of the electron-phonon contribution to the phonon frequencies, which is absent from the resonant bonding viewpoint, indicating the strong coupling of the electronic states along **L–W** line with the soft TO modes.

In summary, we show that the impulsive redistribution of the electronic states near the band edges is consistent with a weakening of the long-range interactions along the <100> cubic direction. This stems from photoexcitation of delocalized carriers from *p*-like resonant bonding states to *p*-like anti-bonding states, thus reducing the propensity for a ferroelectric distortion^5^ and stabilizing the paraelectric state. We show that this effect softens the TA branch while hardening the TO branch near zone centre. These frequency shifts arise in the first principles calculation of photoexcited harmonic force constants, independent of anharmonic effects, indicating that the soft mode behaviour is primarily a consequence of the strong electron-phonon coupling. Our results reconcile the real and reciprocal space pictures of bonding and confirm the importance of electronic states near the band edges in determining the equilibrium structure in resonantly bonded structures^2^,^3^,^4^,^5^.

## Methods

### Experimental details

The reported measurements were performed at the X-ray pump probe instrument of the LCLS X-ray free-electron laser (FEL) using infrared pump pulses (60 fs, 350 μJ, 0.6 eV) generated from an optical parametric amplifier laser and X-ray probe pulses (50 fs, 8.7 keV) with energies selected using a diamond (111) monochromator^34^ leading to a bandwidth of ∼0.5 eV. Both beams approached the sample at a grazing incidence angle (<5°) and the entire sample space was contained within a He-filled chamber to minimize background air scattering. A large area, 2.3 Mpixel Cornell-SLAC Pixel Array Detector with 110 × 110 μm^2^ pixels and a 120 Hz readout rate captured the resulting X-ray diffuse scattering over a wide region of reciprocal space. Preliminary measurements were also taken at the Stanford Synchrotron Radiation Light source (X-ray only) and the Spring-8 Angstrom Compact Free Electron Laser (SACLA) (time-resolved, 1.5 eV optical pump/9 keV X-ray probe).

Diffuse scattering patterns were recorded at a repetition rate of 120 Hz and filtered on a shot-by-shot basis according to fluctuations in both the electron beam energy and X-ray intensity. Images passing the filter constraints were subsequently sorted into 100 fs-wide time delay bins according to the per-shot output of a timing diagnostic tool accurately tracking the relative arrival time between pump and probe^35^. The resulting images in each bin are summed and normalized by the sum of X-ray intensities of the shots corresponding to those images. The reported results stem from the analysis of an average of ∼10,000 shots per time delay bin, which accounting for unused, filtered-out shots, overhead in mechanical movement speeds, and free-electron laser machine downtime, is collected in a matter of several hours in real-time.

Sets of room temperature measurements were taken at two infrared pump fluences, differing by a factor of approximately two. Considering uncertainties in the incidence angle and size of the focused beam, the estimated photoexcited carrier density of the higher pump fluence (the reported data) is 2 × 10^20^ cm^−3^. Other than differences in the amplitude of the differential intensity time-domain responses linear with the pump fluence, no notable variations are seen in a comparison of data from the two fluences. Furthermore, the damage limit of the PbTe samples, from exposure to the combined fluence of both infrared and X-ray laser beams, was assessed at both SACLA and LCLS. The chosen aggregate pump and probe fluence for the measurements was thus kept below this limit. No evidence of sample damage was observed during the experiment.

### Sample growth

Single crystals of PbTe were grown by a modified Bridgman technique with {100} crystallographic orientation at Oak Ridge National Laboratory with n-type carrier concentration of 4 × 10^17^ cm^−3^.

### Geometry and data extraction

We chose the geometric configuration of the experimental setup to simultaneously capture diffuse scattering corresponding to a high-symmetry **Δ** (**Γ** to **X**) line for both an all-even (0 4) and an all-odd (1 3) Brillouin zone (BZ), with select sensitivity to transverse phonon polarization scattering contributions (**q** parallel to the **y**-direction). The geometry was chosen such that the Ewald sphere misses the zone centre (**Γ**) by ∼1% for the (1 3) BZ to prevent damage to the detector by the Bragg diffracted beam.

To enhance the signal-to-noise contrast, multiple neighbouring pixels are binned and averaged together to represent specific coordinates along the subsequent reduced wavevector directions. The **Δ** direction of the (1 3) BZ navigates from **q**∼(0 −0.1 0) to **q**∼(0 −1 0) in (0 −0.05 0) steps, with each coordinate represented by the closest (in reciprocal space) 50 neighbouring pixels, while the **Δ** direction of the (0 4) BZ moves from **q**∼(0 −0.05 0) to **q**∼(0 −0.425 0) in (0 −0.025 0) steps, with each coordinate represented by the closest 10 neighbouring pixels. It should be noted that *q*_x_ and *q*_z_ coordinate values collected near the **Δ** line of the (1 3) BZ remain close to zero throughout the entire reduced wavevector, the same coordinate values deviate slightly from the **Δ** line of the (0 4) BZ. This is due to finite curvature of the Ewald sphere. The deviation from zero for *q*_x_ is ∼−0.003 rlu for each *q* coordinate (segment of binned pixels) moving away from **Γ** and for *q*_z_ is ∼−0.002 rlu. This divergence explains the mismatch with the overlaid dispersion along **Δ** extracted from two times the frequency of the INS results of ref. 28 in Fig. 2d.

To produce the FT-IXS, two-phonon dispersion in Fig. 2b,d, we perform a FT-based analysis to translate the time-domain data into the frequency-domain. Specifically, we apply a Fast Fourier transform algorithm to the time-domain data extracted at each *q* coordinate of a reduced wavevector under investigation. Before employing the algorithm, additional points are padded to each time-domain trace, effectively extending the time delay range to artificially increase the frequency-domain resolution for visual clarity. The padded points in each trace take on the value of the last recorded point in the experimental data. For the reported data, 175 padded points are used for the away from zone centre **Δ** direction traces. The magnitudes of the Fourier coefficients are illustrated in false colour on a base-10 logarithmic scale.

### First principles calculations

The harmonic (second order) and anharmonic (third order) force constants for photoexcited PbTe were calculated using the CDFT approach described in Murray *et al*.^29^. The electronic temperature used in the calculation was 2,000 K, and carriers were assumed to (separately) thermalize within the valence and conduction bands, keeping the total carrier density in the conduction band fixed at 0.5% of the valence electrons. Thus, carrier recombination between conduction and valence bands was assumed to be negligible in the timescale of the experiments. The electronic temperature and the photoexcited carrier concentration values were chosen to give a qualitative picture of the effect of photoexcitation in the experiment. The computed interatomic force constants do not depend strongly on the electronic temperature, and they depend linearly on the photoexcited carrier concentration. We thus interpolated 3rd order force constants for 0.5% carriers using those calculated for 0 and 1%. The second order force constants for non-excited PbTe were calculated using density functional theory (DFT). All the second and third order force constants were calculated from atomic forces using a real-space finite difference supercell approach^36^ and the Phono3py code^37^. All CDFT and DFT calculations were carried out using the ABINIT code^38^ and employing the local density approximation and Hartwigsen–Goedecker–Hutter norm-conserving pseudopotentials. We used the theoretical lattice constant for the local density approximation functional and Hartwigsen–Goedecker–Hutter pseudopotentials for the non-excited material. The lattice constant with the photoexcitation was kept the same since it relaxes on much longer timescales than that at which we track the phonon response. Forces were computed on 216 atom supercells, using an energy cut-off of 15 Ha and 4-shifted 2 × 2 × 2 reciprocal space grids for electronic states. Phonon frequencies, mode eigenvectors, and phonon decay rates were calculated for reduced wave vectors throughout the Brillouin zone as described in He *et al*^39^.

The X-ray diffuse scattering signal arising from phonon squeezing for reduced wave vectors **q** was calculated by taking the overlap 
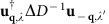
 of the change, 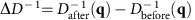, in the inverse dynamical matrix before and after photoexcitation with mode eigenvectors, **u**_**q**,*λ*_ and 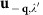 of the photoexcited system. This determines the cross-correlation between modes *λ* and *λ*′ immediately following photoexcitation, which then gives rise to damped oscillations at the sum and difference frequencies, 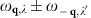, the damping rate of the signal being the average of the energy damping rates of the two modes involved. These damped oscillations of the cross-correlation contribute to the X-ray diffuse scattering signal for momentum transfer, **Q=q+G**, scaled by the polarization factor **Q·u**_**q**,*λ*_ of each mode. Details of this method are in Fahy *et al*.^27^.

### Data availability

The data that support the findings of this study are available from the corresponding authors on request.

## Additional information

**How to cite this article:** Jiang, M. P. *et al*. The origin of incipient ferroelectricity in lead telluride. *Nat. Commun.* 7:12291 doi: 10.1038/ncomms12291 (2016).
